# RED BLOOD CELL DISTRIBUTION WIDTH: WHAT CAN IT TELL US?

**DOI:** 10.1093/geroni/igad104.3559

**Published:** 2023-12-21

**Authors:** Matt Oberdier, Marta Zampino, Michelle Shardell, Majd AlGhatrif, Edward Lakatta, Eleanor Simonsick, Luigi Ferrucci

**Affiliations:** National Institute on Aging, Bethesda, Maryland, United States; University of Maryland, Baltimore, Maryland, United States; University of Maryland School of Medicine, Baltimore, Maryland, United States; NIA/NIH, Baltimore, Maryland, United States; NIH/NIA/ LABORATORY OF CARDIOVASCULAR SCIENCE, Baltimore, Maryland, United States; NIA IRP, Baltimore, Maryland, United States; National Institute on Aging, Baltimore, Maryland, United States

## Abstract

Red blood cell distribution width (RDW), a measure of red blood cell size variability used in evaluating anemia, has been found to predict cardiovascular disease (CVD) morbidity and mortality. RDW increases with age, but the determinants of age-associated elevation in RDW independent of CVD and associated risk factors is limited. Therefore, we examined the association of RDW within the normal range (11.5-15.0%) with age, sex, race, other red blood cell properties, major cardiovascular disease risk factors, and other physiologic data. Data were from 606 men and 627 women (aged 50-95) participating in the Baltimore Longitudinal Study of Aging. Candidate correlates of RDW were included in multiple linear regression models; a reduced model was then obtained by backward selection. RDW was higher with age and female sex. Differences in RDW by sex were explained by hemoglobin, mean corpuscular hemoglobin, and mean corpuscular volume. Further, mean platelet volume (STβ=0.0779, P=0.007), interleukin-6 (STβ=0.0774, P=0.011), erythrocyte sedimentation rate (STβ=0.0746, P=0.028), institutional normalized ratio (STβ=0.0836, P=0.004), thyroid stimulating hormone (STβ=0.0653, P=0.023), and body mass index (STβ=0.1725, P=8.98E-8) were all independently associated with RDW. In the final model, age remained a strong correlate of RDW (STβ=0.2412, P=2.91E-12). In summary, inflammation, metabolic rate, and body habitus independently predict RDW, but do not completely explain its association with age. Future investigation of longitudinal change in RDW with age and its association with these and other factors is needed to advance knowledge on the timing and potential mechanisms of age-associated increases in RDW, and its implication for health outcomes.

